# Evaluation of long-term intravitreal anti-vascular endothelial growth factor injections on renal function in patients with and without diabetic kidney disease

**DOI:** 10.1186/s12882-019-1650-1

**Published:** 2019-12-26

**Authors:** Rachael Ann O’Neill, Patrick Gallagher, Tricia Douglas, Julie-Anne Little, Alexander Peter Maxwell, Giuliana Silvestri, Gareth McKay

**Affiliations:** 1 Centre for Public Health, Queens University Belfast, Belfast, UK; 2 Department of Ophthalmology, Belfast Health and Social Care Trust, Belfast, UK; 3 Optometry & Vision Science Research Group, Biomedical Sciences Research Institute, Ulster University, Coleraine, UK

**Keywords:** Intravitreal anti-vascular endothelial growth factor, Diabetic macular oedema, Renal function

## Abstract

**Background:**

Administering anti-vascular endothelial growth factor (anti-VEGF) by intraocular injection has been shown to have a safe systemic profile. Nevertheless, incidents of acute kidney injury following anti-VEGF injection have been reported. We assessed the long-term effect of multiple intravitreal anti-VEGF injections on measures of renal function in patients with diabetes including rate of change of estimated glomerular filtration rate (eGFR) and urine albumin-to-creatinine ratio (ACR).

**Methods:**

A retrospective review of patients receiving diabetic macular oedema (DMO) treatment was undertaken. Serum creatinine, ACR, number of intravitreal anti-VEGF injections and clinical characteristics were collected from electronic healthcare records (EHR). A co-efficient of eGFR and ACR change with time was calculated over a mean duration of 2.6 years. Regression modelling was used to assess variation in the number of anti-VEGF injections and change in eGFR and ACR.

**Results:**

The EHR of 85 patients with DMO (59% male, 78% type 2 diabetes mellitus [T2DM]) were reviewed. On average, 26.8 intravitreal anti-VEGF injections were given per patient over a mean duration of 31 months. No association between increasing number of anti-VEGF injections and rate of eGFR decline (beta = 0.04, 95% confidence intervals [CI]: − 0.02, 0.09; *p* = 0.22) or ACR change over time (beta = 0.02, CI: − 0.19, 0.23; *p* = 0.86) was detected, following adjustment for hypertension, cerebrovascular disease, T2DM, and medications taken.

**Conclusion:**

Our data suggests regular long-term intravitreal VEGF inhibition does not significantly alter the rate of change in eGFR and/or ACR with increasing number of treatment injections.

## Background

Vascular endothelial growth factor (VEGF) inhibitors have transformed the therapeutic management of several retinal ophthalmic conditions. By improving visual acuity, they have surpassed the ability of laser photocoagulation to limit visual deterioration [1]. Despite their current extensive use for the treatment of diabetic macular oedema (DMO), neovascular age-related macular degeneration and retinal vein occlusion, their initial primary application was as an intravenous chemotherapeutic adjunct in the form of bevacizumab, for the treatment of solid tumours, including breast, colorectal and lung cancer [2, 3].

In addition to its role in the eye, VEGF plays a crucial part in maintaining normal renal function. VEGF released from podocytes interacts with VEGF receptor 2 on glomerular capillaries and promotes integrity of endothelial fenestrations and resultant glomerular barrier function [4]. Loss of podocyte dependent VEGF expression in gene knockout mice resulted in proteinuria, hypertension and renal thrombotic microangiopathy [5]. A systematic review and meta-analysis of 1850 patients across seven clinical trials revealed a significant dose dependent increase in risk associated with hypertension and proteinuria in those receiving intravenous bevacizumab [6]. From 2005 to 2012, there were more than 50 reported cases of renal thrombotic microangiopathy, increased proteinuria and antibody-mediated kidney transplantation rejection following intravenous administration of VEGF inhibitors [3, 7, 8]. These findings suggest that when administered systemically at high dosage, VEGF inhibitors may have a serious adverse effect on renal function.

Sustained hyperglycaemia secondary to diabetes mellitus (DM) has been shown to activate abnormal metabolic pathways that trigger a complex cascade of inflammatory and vasogenic responses in the eye [1]. VEGF is a major driver in the pathophysiology of DMO as it promotes retinal angiogenesis and capillary hyper-permeability that can disrupt the internal blood retinal barrier, resulting in leakage of fluid into the retinal tissue. DMO is one of the leading causes of vision loss in adults [9]. Given the key role that VEGF plays in the pathogenesis of DMO, intravitreal VEGF inhibitors have become integral in the treatment of this condition.

Use of VEGF inhibition therapy as an ophthalmic therapeutic involves local administration into the vitreous humour by intra-ocular injection with the dosage used approximately 400 times lower than that used in oncology [10, 11]. Aflibercept and ranibizumab, two more recent anti-VEGF agents with different structures and pharmacokinetic profiles, were developed specifically for intravitreal use [12]. Such adaptations have improved the systemic safety profile of intravitreal anti-VEGF in the general population, although longer term further evaluation of systemic safety is ongoing.

Intravitreal ranibizumab (IVR) exists as a human monoclonal Fab antibody fragment with a molecular weight of 48 kDa and binds to all isoforms of VEGF-A [13]. Intravitreal aflibercept (IVA) is a human recombinant fusion protein with a molecular weight of 115 kDa, which binds to all isoforms of VEGFA, VEGFB and placental growth factor [7]. Although the pharmacokinetic profiles of ranibizumab and aflibercept are notably different, studies have shown that both traverse the blood retinal barrier and enter the systemic circulation, reducing circulating levels of VEGF [11–15]. There have been cases reports of unilateral IVR and IVA injection having a significant therapeutic effect on the contralateral eye [16–20].. These examples indicate there are systemic responses to intravitreal anti-VEGF agents. Furthermore, there have been several cases of acute kidney injury (AKI) reported in patients with chronic kidney disease (CKD) and renal allograft dysfunction with increased hypertension and proteinuria, following intravitreal anti-VEGF injection [8, 20–24]. Despite established incidents of acute renal impairment following anti-VEGF therapy, studies directly investigating their impact on long-term kidney function have been limited.

A pooled analysis of 751 population-based studies reported a global increase in the number of adults with DM from 108 million in 1980 to 422 million in 2014 [25]. It is predicted that rapid increase in DM prevalence will result in a parallel increase in diabetic microvascular complications including diabetic retinopathy and nephropathy [26], reinforcing the need for stringent safety evaluation of intravitreal anti-VEGF therapies. This retrospective observational audit evaluated the effects of cumulative anti-VEGF exposure, following repeated intra-ocular injections, on estimated glomerular filtration rate (eGFR) and urine albumin-to-creatinine ratio (ACR) in patients with DMO, to evaluate variation in renal function associated with long-term intraocular anti-VEGF injections.

## Methods

This was a retrospective, cohort, observational study using electronic healthcare records to access information on patients with DMO receiving intravitreal anti-VEGF treatment in the Belfast Health and Social Care Trust. This study received approval by the Office for Research Ethics Committee Northern Ireland (MREC Reference: 14/NI/1132).

Serum creatinine measurements (μmol/L) and ACR (mg/mmol) were collected from the Northern Ireland Electronic Care Record (ECR) system. Each eGFR measurements was calculated using the Chronic Kidney Disease Epidemiology Collaboration equation (CKD-EPI). In this study, participants received aflibercept, ranibizumab or both throughout the course of their treatment. The number of aflibercept, ranibizumab and total intravitreal anti-VEGF injections was recorded for each participant. Changes in eGFR and ACR over time were calculated using multiple eGFR and ACR measurements. These included a measure of renal function before the start of anti-VEGF therapy and after the defined injection period. Data was collected on demographic factors, glycaemic parameters and clinical variables including co-morbidities and medications.

This study included patients who were administered their first to last recorded anti-VEGF injections between 25th April 2012 and 22nd January 2018. For inclusion, each patient was required to have renal function measurements prior to their first anti-VEGF injection and after their last injection was administered. Patients were excluded on the basis of insufficient number of renal function measurements or if they experienced an acute decline in eGFR or rapid increase in ACR. Patients with diabetic kidney disease (DKD) can be classified depending on their level of kidney function (eGFR) and the amount of protein present in the urine (ACR). This information forms the basis of DKD staging which is useful for planning follow up and management. Individuals were classified as ‘No DKD’ if they had an ACR < 3 mg/mmol and an eGFR > 60 mL/min/1.73 m^2^. Study participants with an ACR > 3 mg/mmol or an eGFR < 60 mL/min/1.73 m^2^ were classified as DKD.

### Statistical analysis

Independent samples T-tests, chi squared or Fishers exact tests were used to compare the distribution of demographic factors, glycaemic parameters and clinical variables between patients with DKD and those without DKD. Covariates significantly associated with a diagnosis of DKD were adjusted for in subsequent linear regression modelling. Simple and multiple linear regression models were used to generate beta estimates (β) and 95% confidence intervals (CI) for the total number of intravitreal anti-VEGF injections against the change in eGFR and ACR over time. *P* < 0.05 was considered statistically significant.

In cases where ACRs were not present as absolute values (e.g. < 3 mg/mmol), arbitrary values were used to facilitate slope calculation. A previous study has demonstrated that the median ACR value for patients with an ACR < 3 mg/mmol was 1.06 mg/mmol and we used this as an arbitrary value for ACR values categorised as < 3 mg/mmol on the ECR [27].

## Results

Data was collected on 90 patients undergoing regular intravitreal anti-VEGF treatment for DMO in the Belfast Health and Social Care Trust. Although, episodes of acute kidney injury following anti-VEGF injection have been reported previously, in order to evaluate the long-term effect of intravitreal anti-VEGF treatment on renal function and limit potential confounding from co-morbidities, five patients were excluded because an obvious reported co-morbidity led directly to an acute decline in renal function. A total of 42 participants were classified as ‘No DKD’ controls and 43 individuals were classified as ‘DKD cases’. Study cohort characteristics, co-morbidities and glycaemic parameters are summarised (Table 1). The mean HbA1c was 67.3, standard deviation (SD) = 16.1 mmol/mol and mean duration of DM was 16.5, SD = 11.4 years. Additionally, 84 and 77% of participants had hypertension and hyperlipidaemia, respectively.

**Table 1:** Participant summary characteristics

Characteristic	All (*n* = 85)	No DKD (*n* = 42)	DKD (*n* = 43)	*P* value
Mean Baseline eGFR (ml/min/1.73 m2, SD)	75.0 ± 21.4	83.8 ± 13.3	66.5 ± 24.4	< 0.01
Mean Baseline ACR (mg/mmol, SD)	17.9 ± 62.1	1.0 ± 0.67	34.4 ± 84.6	0.01
Male, n (%)	50 (58.8)	24 (57.1)	26 (60.5)	0.76
Age at 1st injection (years, SD)	64.4 ± 9.4	62.9 ± 7.7	65.8 ± 10.6	0.16
Type 2 Diabetes mellitus, n (%)	66 (77.6)	30 (71.4)	36 (83.7)	0.17
Mean Duration of diabetes (years, SD)	16.5 ± 11.4	16.2 ± 12.4	16.7 ± 10.6	0.85
Mean HbA1c (mmol/mol, SD)	67.3 ± 16.1	65.7 ± 13.1	68.9 ± 18.5	0.36
Hypertension, n (%)	71 (83.5)	30 (71.4)	41 (95.3)	< 0.01
Hyperlipidaemia, n (%)	65 (76.5)	31 (73.8)	34 (79.1)	0.57
Coronary Heart Disease or Heart Failure, n (%)	31 (36.5)	11 (26.2)	20 (46.5)	0.05
Cerebrovascular Disease, n (%)	12 (14.1)	2 (4.8)	10 (23.3)	0.03
Never smoked, n (%)	53 (62.4)	28 (66.6)	25 (58.1)	0.04
Angiotensin Converting Enzyme Inhibitor, n (%)	41 (48.2)	19 (45.2)	22 (51.2)	0.59
Angiotensin Receptor Antagonist, n (%)	14 (16.5)	6 (14.3)	8 (18.6)	0.59
Calcium Channel Blocker, n (%)	34 (40.0)	12 (28.6)	22 (51.2)	0.03
Thiazide Diuretic, n (%)	7 (8.2)	2 (4.8)	5 (11.6)	0.43
Spironolactone, n (%)	3 (3.5)	0 (0.0)	3 (7.0)	0.24
Loop Diuretic, n (%)	13 (15.3)	1 (2.4)	12 (27.9)	< 0.01
Beta Blocker, n (%)	26 (30.6)	8 (19.0)	18 (41.9)	0.02
Statin, n (%)	68 (80.0)	32 (76.2)	36 (83.7)	0.39
Metformin, n (%)	53 (62.4)	24 (57.1)	29 (67.4)	0.33
Aspirin, n (%)	39 (45.9)	20 (47.6)	19 (44.2)	0.75
Alpha Blockers, n (%)	11 (12.9)	4 (9.5)	7 (16.3)	0.52
Clopidogrel, n (%) Proton Pump Inhibitor, n (%)	12 (14.1) 24 (28.2)	7 (16.6) 10 (23.9)	5 (11.6) 14 (32.6)	0.51 0.37

Values provided are n (%) for categorical variables and mean ± SD for continuous variables

Abbreviations: DKD, diabetic kidney disease; eGFR, estimated glomerular filtration rate; ACR, albumin-to-creatinine ratio; HbA1c, glycated haemoglobin; SD, standard deviation

The eGFR data met the assumptions of linear regression including normal distribution, homoscedasticity and absence of multicollinearity. However, ACR data was skewed lacking normal distribution and homoscedasticity. Importantly, absence of multicollinearity remained. Log transformation of ACR data did not improve the distribution curve and as a result, no log transformation was performed.

Participants demonstrated a decline in eGFR from a mean baseline of 75 mL/min/1.73 m^2^ to a mean follow-up eGFR of 65.9 mL/min/1.73 m^2^ with a mean rate of decline of 2.6 mL/min/1.73 m^2^ /year (Table 2). On average, participants received 26.8 ± 13.2 intravitreal anti-VEGF injections, which included 16.6 ± 10.0 ranibizumab and 10.1 ± 6.0 aflibercept, over a mean duration of 31 months (2.6 years). In an unadjusted linear regression analysis, the rate of change of eGFR over time was not significantly associated with the number of intravitreal anti-VEGF injections (β = 0.04, CI: − 0.02, 0.09; *p* = 0.21) and remained non-significant following adjustment for T2DM, cerebrovascular disease (CVD), hypertension and treatment with proton pump inhibitors (β = 0.04, CI: − 0.02, 0.09; *p* = 0.22).

**Table 2:** Intravitreal anti-VEGF injections and renal function

Clinical Variable	All (n = 85)	No DKD (n = 42)	DKD (n = 43)	P value
Mean No. anti-VEGF injections (SD)	26.8 ± 13.2	28.6 ± 12.5	25.0 ± 13.8	0.22
Mean No. ranibizumab injections (SD)	16.6 ± 10.0	17.2 ± 10.4	16.0 ± 9.7	0.59
Mean No. aflibercept injections (SD)	10.1 ± 6.0	11.2 ± 4.5	9.0 ± 7.0	0.09
Mean baseline eGFR (mL/min/1.73 m^2^, SD)	75.0 ± 21.4	83.8 ± 13.3	66.5 ± 24.4	< 0.01
Mean follow-up eGFR (mL/min/1.73 m^2^, SD)	65.9 ± 22.9	75.7 ± 15.9	57.1 ± 24.6	< 0.01
Mean change in eGFR (mL/min/1.73 m^2^, %)	−8.7 (12.1)	−8.0 (9.5)	−9.4 (14.1)	0.56
Mean eGFR slope (mL/min/1.73 m^2^, SD)	−2.6 ± 3.5	−2.7 ± 3.4	- 2.5 ± 3.6	0.84
Mean baseline ACR (mg/mmol, SD)	17.9 ± 62.1	1.0 ± 0.67	34.4 ± 84.6	0.01
Mean follow-up ACR (mg/mmol, SD)	18.8 ± 48.5	1.8 ± 2.6	35.4 ± 64.3	< 0.01
Mean change in ACR (mg/mmol, %)	+ 0.94 (5.0)	+ 0.86 (80)	1.0 (2.9)	0.99
Mean ACR slope (mg/mmol, SD)	0.7 ± 12.3	0.2 ± 0.7	1.3 ± 17.4	0.69

Abbreviations: DKD, diabetic kidney disease; eGFR, estimated glomerular filtration rate; ACR, albumin-to-creatinine ratio; SD, standard deviation

As expected, participants with DKD had a significantly lower mean baseline eGFR of 66.5 ± 24.4 mL/min/1.73 m^2^ compared to 83.8 ± 13.3 mL/min/1.73 m^2^ /year in patients without DKD (*p* < 0.01). Additionally, patients with DKD also had significantly lower follow-up eGFR at 57.1 ± 24.6 mL/min/1.73 m^2^ compared to 75.7 ± 15.9 mL/min/1.73 m^2^ (p < 0.01). Patients with DKD did not have a greater rate of eGFR decline (− 2.5 ± 3.6 mL/min/1.73 m^2^ /year) compared to individuals without DKD (− 2.7 ± 3.4 mL/min/1.73 m^2^ /year).

Study participants had increased ACR from a mean baseline value of 17.9 ± 62.1 mg/mmol to a mean follow-up ACR of 18.8 ± 48.5 mg/mmol with a rate of increase of 0.7 ± 12.3 mg/mmol/year. In an unadjusted analysis the rate of change of ACR over time was not significantly associated with the number of intravitreal anti-VEGF injections increased (β = 0.01, CI: − 0.19, 0.22; *p* = 0.91) and remained non-significant following adjustment for T2DM, CVD, and treatment with beta blockers and proton pump inhibitors (β = 0.02, CI: − 0.19, 0.23; *p* = 0.86).

Participants with DKD had significantly higher mean baseline ACR of 34.4 ± 84.6 mg/mmol compared to 1.0 ± 0.67 mg/mmol in patients without DKD (*p* < 0.01). Additionally, participants with DKD had significantly higher ACR at follow-up 35.4 ± 64.3 mg/mmol compared to 1.8 ± 2.6 mg/mmol (p < 0.01).

Across all participants, the mean number of ranibizumab injections received by those with DKD was 16.0 ± 9.7 injections compared to 17.2 ± 10.5 in those without DKD (*p* = 0.59). The mean number of aflibercept injections received by participants with DKD was 9.0 ± 7.0 injections compared to 11.2 ± 4.5 injections in those without DKD (*p* = 0.09).

## Discussion

There is a strong correlation between the progression of retinal and renal microvascular complications as a consequence of long-term hyperglycaemia. This highlights the importance of evaluating the long-term efficacy and renal safety of intravitreal anti-VEGF agents [28]. This study demonstrates that long-term intravitreal ranibizumab and aflibercept injections for the treatment of DMO does not significantly alter the rate of change in eGFR or ACR over time, therefore adding further support to the safety profile of intravitreal anti-VEGF.

A retrospective study of renal safety following acute anti-VEGF exposure showed no significant change in mean eGFR and no episodes of acute kidney injury, following a single intravitreal anti-VEGF injection of ranibizumab, aflibercept or bevacizumab, in a cohort of 69 patients with DM and CKD [29]. However, the study by Kameda and colleagues did not consider the potential cumulative effects of intravitreal anti-VEGF exposure on renal function. In estimating the change in eGFR and ACR prior to and over the anti-VEGF treatment period, the current study was able to evaluate the longitudinal effect of VEGF inhibition on renal outcomes. Our study found no significant association between increased intravitreal anti-VEGF exposure and eGFR or ACR, over an average duration of 31 months.

Both a comprehensive review (4203 patients from 10 studies) and meta-analysis (10,300 patients from 22 studies) investigated the systemic safety profile of IVA and IVR respectively, in DMO, neovascular age-related macular degeneration and retinal vein occlusion, by pooling data from existing randomised controlled trials but found no difference in the incidence of adverse systemic events between either intravitreal anti-VEGF treatment and placebo [30, 31]. Adverse events recorded were not considered to be attributable to the study drug. These results further support the findings from individual randomised trials demonstrating a consistent safety profile, including no adverse impact on renal function, across a range of retinal disorders.

It is important to highlight that the clinical trials investigating IVR and IVA in DMO were not designed or powered to evaluate differences in low frequency systemic events, mainly as a consequence of their small sample sizes. Therefore, a firm conclusion on the systemic safety profile of intravitreal anti-VEGF is limited. Larger prospective studies with a longer follow up period and sufficient power to assess low frequency systemic adverse effects are required. A greater focus on the systemic safety of intravitreal anti-VEGF in high-risk groups is also needed. A population based, nested case-control study including 91,000 participants assessed post-marketing data on intravitreal anti-VEGF injections and found no significant increased risk of stroke, myocardial infarction, venous thromboembolism or congestive heart failure [32]. While the study did not consider risk of AKI or CKD, a similar post-marketing population based study would be of value in assessing long-term renal safety. Additionally, existing clinical trial data rely on detection of AKI events rather than more subtle changes in markers of renal function associated with CKD. Drugs that accelerate the decline in kidney function without registering as AKI or CKD are important given the increased risk of micro-vasculopathy in diabetic patients and often parallel pathophysiological changes in retinal and renal vasculature.

The Diabetic Retinopathy Clinical Research Network measured baseline and 52-week follow-up urinary ACR in 654 patients receiving ranibizumab, aflibercept or bevacizumab. On average, each patient had 9–10 injections during the treatment period. Across all three treatment groups, over 77% of patients maintained their baseline urinary ACR, while 10–16% of patients experienced a worsening of ACR by the 52-week follow up period, with more than 7% of patients experiencing an improvement in ACR. In the absence of a control group no definitive assessment could be made on the influence of anti-VEGF treatment. However, intravitreal anti-VEGF treatment did not appear to increase the risk of developing or worsening proteinuria [33].

In our study 54, 34 and 12% of patients had a baseline ACR < 3, 3–30 and > 30 mg/mmol, respectively, with no significant change detected over the 2.6 year treatment period. In comparison, the percentage of participants with an eGFR < 60 mL/min/1.73 m^2^ increased from 26% at baseline to 39% at follow up, following an average duration of 2.6 years of anti-VEGF treatment. The difference observed for both renal markers highlights the variation in the sensitivity of their measurement outcomes and the importance of monitoring both in diabetic populations.

In this study, 66 patients with DMO had T2DM and 19 patients had type 1 DM (T1DM). The mean baseline eGFR for patients with T2DM was lower at 74.1 mL/min/1.73 m^2^ compared to an eGFR of 78.1 mL/min/1.73 m^2^ for patients with T1DM. The mean follow-up eGFR was also lower for T2DM patients with an eGFR of 64.9 mL/min/1.73 m^2^ compared to T1DM with an eGFR of 71.3 mL/min/1.73 m^2^. The mean rate of eGFR decline was 2.9 mL/min/1.73 m^2^ /year compared to 1.6 mL/min/1.73 m^2^ /year for T2DM and T1DM respectively. Eighty four per cent of patients with T2DM had a diagnosis of CKD compared to 16% of patients with T1DM. Our findings reflect those from a large US study which showed a significantly higher prevalence of CKD in T2DM compared to T1DM patients (44% vs. 32% respectively, *p* < 0.001) [34].

There are a number of limitations with our study including the inability to perform a sensitivity analysis to assess the relative contributions of IVR and IVA on change in renal function over time. However, a secondary analyses of a randomised comparative effectiveness trial, known as Protocol T, carried out by the Diabetic Retinopathy Clinical Research Network, showed no significant difference in renal function as assessed by urinary ACR over a 52 week follow-up period between patients who received intravitreal ranibizumab, aflibercept or bevacizumab for the treatment of DMO [33]. In addition, the limited sample size of 85 patients may have been insufficient to detect associations with change in renal function. This was a retrospective observational study, which prevented us performing a priori power analysis. In addition, we did not differentiate between patients in receipt of unilateral or bilateral injections which may influence the rate of adverse events observed [35–37]. Moreover, given our study did not provide a direct comparison between participants with DMO and those not undergoing VEGF inhibition therapy, it was not possible to determine whether the rate of renal decline over time differed between those in receipt of treatment and those who were not. Furthermore, due to the high prevalence of co-morbidities in diabetic populations, analysis of individuals with neovascular age-related macular degeneration may provide a more opportunistic comparison and a reduced risk of residual confounding.

Despite these limitations, our study had several strengths. In collecting prospective eGFR/ACR data we were able to assess long-term changes in renal function that would not have been reported as an adverse event. Additionally, we collected data on a wide range of co-morbidities and glycaemic parameters, which allowed for appropriate adjustment of potential confounding factors. We used the CKD-EPI equation rather than the Modification of Diet in Renal Disease equation to calculate estimated glomerular filtration rates. The CKD-EPI equation is generally considered to be a better predictor of renal function, particularly at higher eGFR values [38].

## Conclusions

This study supports the previously demonstrated effective renal safety profile of intravitreal anti-VEGF in patients with DMO. Regular long-term intravitreal VEGF inhibition does not significantly alter the rate of change in eGFR and/or ACR with increasing number of treatment injections. The long-term assessment of renal function provides additional evaluation and detection of subtle changes in eGFR and ACR that may not present clinically as adverse events. Larger prospective and post-marketing trials, using renal markers including eGFR, ACR and Cystatin C, as well as assessing incidence of AKI and CKD, are required to strengthen the renal safety of intravitreal anti-VEGF treatment modalities. A greater focus on at-risk groups such as those with CKD is required.

## Abbreviations

ACR: albumin-to-creatinine ratio
AKI: acute kidney injury
Anti-VEGF: anti-vascular endothelial growth factor
CKD: chronic kidney disease
CKD-EPI: chronic kidney disease epidemiology collaboration equation
CVD: cerebrovascular disease
DKD: diabetic kidney disease
DM: diabetes mellitus
DMO: diabetic macular oedema
eGFR: estimated glomerular filtration rate
EHR: electronic healthcare records
HbA1c: glycated haemoglobin
IVA: intravitreal aflibercept
IVR: intravitreal ranibizumab
T1DM: type 1 diabetes mellitus
T2DM: type 2 diabetes mellitus
UACR: urinary albumin-to-creatinine ratio
